# Dataset on the new recipe for the preparation of nanoporous carbon nanorods using resorcinol-formaldehyde xerogels

**DOI:** 10.1016/j.dib.2018.03.087

**Published:** 2018-03-26

**Authors:** Ahmed Awadallah-F, Shaheen A. Al-Muhtaseb

**Affiliations:** Department of Chemical Engineering, Qatar University, PO Box 2713, Doha, Qatar

**Keywords:** Resorcinol, Formaldehyde, Xerogel, Porous carbon nanorods

## Abstract

The data presented in this article are related to the research article entitled “Novel controlled synthesis of nanoporous carbon nanorods from resorcinol-formaldehyde xerogels" (Awadallah-F and Al-Muhtaseb, 2017) [1]. This article describes the novel controlled approach of nanoporous carbon nanorods synthesis from resorcinol/formaldehyde xerogels. The field dataset is made publicly available to enable critical or extended analyzes.

**Specifications Table**

**Table:** 

**Subject area**	Gel Chemistry
**More specific subject area**	Xerogel for carbon nanorods
**Type of data**	Figures
**How data was acquired**	Micromeritics ASAP2420 and XRD
**Data format**	Raw data collection and analysis
**Experimental factors**	N_2_ gas adsorption/desorption isotherms and XRD analysis of samples.
**Experimental features**	The relationship between the gelation temperature and xerogels-based-carbon nanorods
**Data source location**	Doha, Qatar 25.375N,51.4905E
**Data accessibility**	The data are available with this article
**Related research article**	It is a companion to the article under title of "Novel Controlled Synthesis of Nanoporous Carbon Nanorods from Resorcinol-Formaldehyde Xerogels"

**Value of the data**

• The data presents the adsorption/desorption isotherms of N_2_ gas on xerogel carbon nanorods.
• The data presents the XRD patterns of two samples xerogel-based carbon nanorods that were prepared at 70 °C and 85 °C.
• Authors publish these data in purpose of consulting in future research, teaching and acquiring innovative knowledge.

## 1 Data

**Fig. 1:**
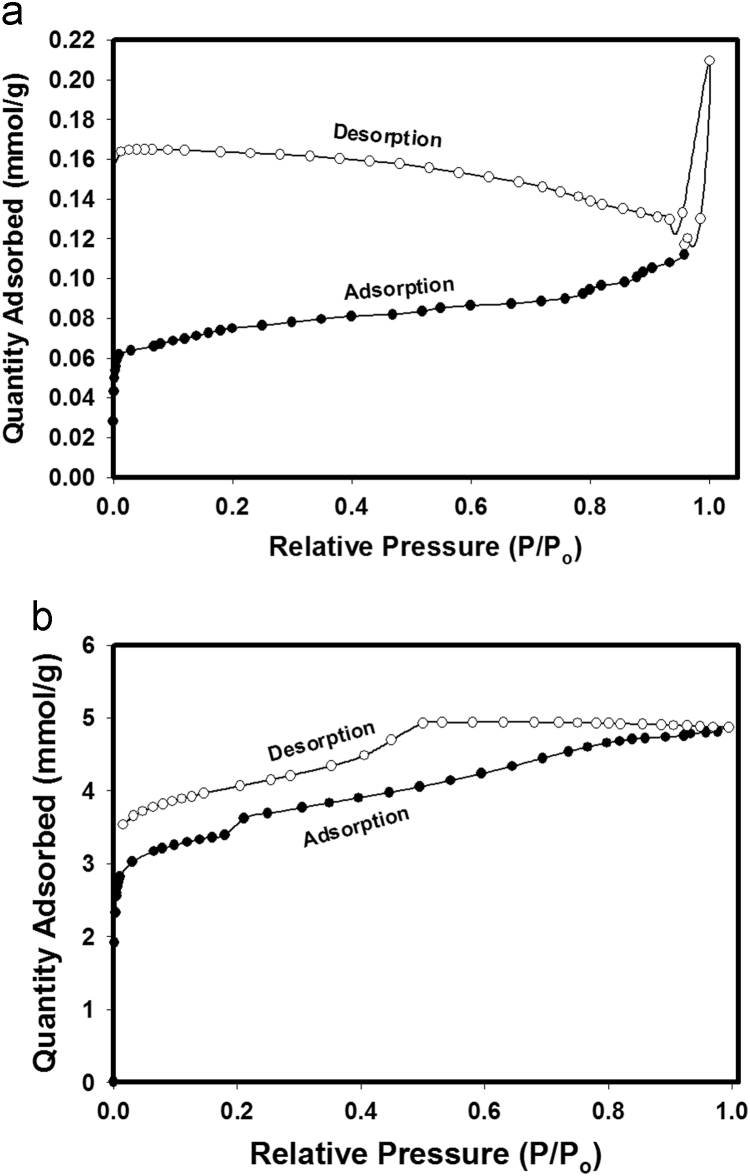
Adsorption/desorption isotherms of nitrogen at 77 K on (a) RF-ACX-1 and (b) RF-ACX-2 samples.

**Fig. 2:**
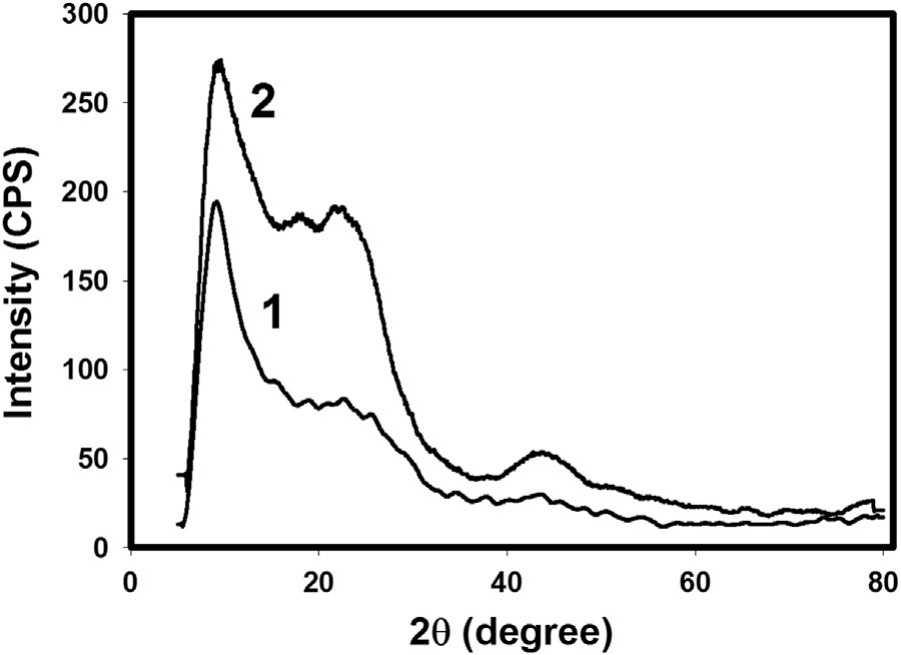
XRD patterns of RF-ACX-1 and RF-ACX-2 (curves 1 and 2, respectively).

The dataset of this article is information on the novel controlled synthesis of nanoporous carbon nanorods by using resorcinol-formaldehyde xerogels as the main reactants. Fig. 1, Fig. 2 show adsorption/desorption isotherm curves and XRD patterns, respectively.

## 2 Experimental design, materials and methods

### 2.1 Gel synthesis

In order to prevent the dehydration of the gels formed, and to increase their cross-linking in the same time, 2% acetic acid was poured upon the gels surfaces after their solidification. Then, the vials were removed from the oven and allowed to cool to room temperature. The remnant solutions above the cured gels were then decanted and exchanged with acetone at room temperature. The samples were left in acetone at room temperature, and the remaining acetone is replaced with fresh acetone daily for 3 days. After the third day of solvent exchange, the cured gels and the accompanying fresh acetone were placed in the oven at 50 °C, and were kept for 2 days to dry at that condition. Upon drying at atmospheric conditions, the samples were carbonized in a flowing stream of nitrogen gas, and then activated physically in a flowing stream of carbon dioxide gas. The resulting resorcinol-formaldehyde activated carbon xerogels (RF-ACXs) prepared at the gelation temperatures of 70 °C and 85 °C are coded as RF-ACX-1 and RF-ACX-2, respectively [1], [2].

### 2.2 Carbonization and activation of xerogel sample

The dried RF xerogel was placed in a ceramic boat inside a programmable electric-heated tube furnace (Nabertherm GmbH), with a continuous flow of nitrogen (100 cm^3^/min). The furnace was first maintained at room temperature for 30 min to make sure that the air is completely purged with the flowing nitrogen. Then, the furnace was heated up to a temperature of (500 °C) with a heating rate of 10 °C/min. The sample was maintained at 500 °C K for 3 h, and then allowed to cool to room temperature while passing nitrogen. The resulting carbon xerogel was then activated in the same tube furnace with CO_2_ flow (150 cm^3^/min) instead of nitrogen, heating the sample again with a rate of 10 °C/min to 700 °C, maintaining this temperature for 1 h, and then allowing the sample to cool down to room temperature while passing CO_2_ [2].

### 2.3 Characterization

The pore structures of RF-ACXs were measured using the adsorption/desorption isotherms of nitrogen at 77 K (see Fig. 1) via a Micromeritics ASAP2420^®^ accelerated surface area and porosimetry system with an enhanced micropore capability (utilizing a 1-Torr pressure transducer to increase precision in the micropore range). Prior to adsorption measurements, the samples were regenerated in-situ for 8 h at 150 °C under high vacuum (1×10^−6^ mbar). The pore size distributions were obtained by density functional theory (DFT) [3]. Fourier transform infrared (FTIR) spectra were measured with ATI Genesis Series FTIR spectrophotometer using KBr pellet in wave numbers between 4000 and 400 cm^−1^ to ascertain the structure of RF-ACX samples. Raman spectroscopy (DXR Raman microscope, thermofisher scientific Madison, USA). The morphology of RF-ACXs was observed with a FEI Nova™ NanoScanning Electron Microscope 450 (Nova NanoSEM). The chemical compositions of samples were investigated by an elemental CHNS/O analyzer (2400, Series II, Perkin Elmer). Wide-angle X-ray diffraction (WAXD) measurements were conducted in the reflection mode at room temperature using a Bruker-D2 Phaser. The Cu Kα radiation (*λ*=1.54 Å) source was operated at a voltage of 50 kV and a current of 40 mA. The 2*θ* scan data were collected at 0.02° intervals over the range of 5–80°, and at a scan speed of 0.02(2 h) min^−1^. The XRD patterns of RF-ACX-1 and RF-ACX-2 are shown in Fig. 2.
